# Supplementary material for: Pyruvate Dehydrogenase Kinase 1 inhibition mediated oxidative phosphorylation enhancement in cartilage promotes osteoarthritis progression
Source: BMC Musculoskelet Disord. 2023 Jul 20;24:597. doi: 10.1186/s12891-023-06585-6 (PMC10357736; doi:10.1186/s12891-023-06585-6)
Supplement: Supplementary file 1 — Additional file 1. [file 12891_2023_6585_MOESM1_ESM.zip › KEGG.Gsea.1653622432464/KEGG_NUCLEOTIDE_EXCISION_REPAIR.html]

Details for gene set KEGG\_NUCLEOTIDE\_EXCISION\_REPAIR[GSEA]

|  || Dataset | 55457\_collapsed\_to\_symbols.cls.cls #OA\_versus\_normal.cls.cls #OA\_versus\_normal\_repos |
| Phenotype | cls.cls#OA\_versus\_normal\_repos |
| Upregulated in class | OA |
| GeneSet | KEGG\_NUCLEOTIDE\_EXCISION\_REPAIR |
| Enrichment Score (ES) | 0.49285778 |
| Normalized Enrichment Score (NES) | 1.9538083 |
| Nominal p-value | 0.0 |
| FDR q-value | 0.0042137792 |
| FWER p-Value | 0.027 |
Table: GSEA Results Summary

  

Fig 1: Enrichment plot: KEGG\_NUCLEOTIDE\_EXCISION\_REPAIR      
 Profile of the Running ES Score & Positions of GeneSet Members on the Rank Ordered List

  

| SYMBOL | TITLE | RANK IN GENE LIST | RANK METRIC SCORE | RUNNING ES | CORE ENRICHMENT || 1 | RPA3 | replication protein A3 [Source:HGNC Symbol;Acc:HGNC:10291] | 13 | 1.159 | 0.0686 | Yes |
| 2 | CETN2 | centrin 2 [Source:HGNC Symbol;Acc:HGNC:1867] | 130 | 0.875 | 0.1122 | Yes |
| 3 | RPA2 | replication protein A2 [Source:HGNC Symbol;Acc:HGNC:10290] | 339 | 0.727 | 0.1397 | Yes |
| 4 | RBX1 | ring-box 1 [Source:HGNC Symbol;Acc:HGNC:9928] | 348 | 0.724 | 0.1826 | Yes |
| 5 | ERCC8 | "ERCC excision repair 8, CSA ubiquitin ligase complex subunit [Source:HGNC Symbol;Acc:HGNC:3439]" | 506 | 0.656 | 0.2098 | Yes |
| 6 | RFC3 | replication factor C subunit 3 [Source:HGNC Symbol;Acc:HGNC:9971] | 570 | 0.633 | 0.2429 | Yes |
| 7 | RPA1 | replication protein A1 [Source:HGNC Symbol;Acc:HGNC:10289] | 571 | 0.632 | 0.2809 | Yes |
| 8 | POLD1 | "DNA polymerase delta 1, catalytic subunit [Source:HGNC Symbol;Acc:HGNC:9175]" | 811 | 0.566 | 0.2963 | Yes |
| 9 | POLD4 | "DNA polymerase delta 4, accessory subunit [Source:HGNC Symbol;Acc:HGNC:14106]" | 820 | 0.564 | 0.3296 | Yes |
| 10 | CDK7 | cyclin dependent kinase 7 [Source:HGNC Symbol;Acc:HGNC:1778] | 834 | 0.560 | 0.3622 | Yes |
| 11 | RFC4 | replication factor C subunit 4 [Source:HGNC Symbol;Acc:HGNC:9972] | 897 | 0.543 | 0.3900 | Yes |
| 12 | RFC1 | replication factor C subunit 1 [Source:HGNC Symbol;Acc:HGNC:9969] | 931 | 0.531 | 0.4193 | Yes |
| 13 | GTF2H5 | general transcription factor IIH subunit 5 [Source:HGNC Symbol;Acc:HGNC:21157] | 1032 | 0.511 | 0.4422 | Yes |
| 14 | RFC2 | replication factor C subunit 2 [Source:HGNC Symbol;Acc:HGNC:9970] | 1247 | 0.467 | 0.4537 | Yes |
| 15 | PCNA | proliferating cell nuclear antigen [Source:HGNC Symbol;Acc:HGNC:8729] | 1557 | 0.419 | 0.4548 | Yes |
| 16 | XPA | "XPA, DNA damage recognition and repair factor [Source:HGNC Symbol;Acc:HGNC:12814]" | 1615 | 0.411 | 0.4751 | Yes |
| 17 | ERCC4 | "ERCC excision repair 4, endonuclease catalytic subunit [Source:HGNC Symbol;Acc:HGNC:3436]" | 1696 | 0.400 | 0.4929 | Yes |
| 18 | DDB1 | damage specific DNA binding protein 1 [Source:HGNC Symbol;Acc:HGNC:2717] | 2302 | 0.326 | 0.4654 | No |
| 19 | ERCC1 | "ERCC excision repair 1, endonuclease non-catalytic subunit [Source:HGNC Symbol;Acc:HGNC:3433]" | 2948 | 0.255 | 0.4306 | No |
| 20 | DDB2 | damage specific DNA binding protein 2 [Source:HGNC Symbol;Acc:HGNC:2718] | 3257 | 0.225 | 0.4202 | No |
| 21 | XPC | "XPC complex subunit, DNA damage recognition and repair factor [Source:HGNC Symbol;Acc:HGNC:12816]" | 3278 | 0.222 | 0.4320 | No |
| 22 | ERCC2 | "ERCC excision repair 2, TFIIH core complex helicase subunit [Source:HGNC Symbol;Acc:HGNC:3434]" | 3544 | 0.198 | 0.4233 | No |
| 23 | CUL4B | cullin 4B [Source:HGNC Symbol;Acc:HGNC:2555] | 3783 | 0.179 | 0.4155 | No |
| 24 | POLE3 | "DNA polymerase epsilon 3, accessory subunit [Source:HGNC Symbol;Acc:HGNC:13546]" | 3934 | 0.162 | 0.4136 | No |
| 25 | CCNH | cyclin H [Source:HGNC Symbol;Acc:HGNC:1594] | 4120 | 0.144 | 0.4078 | No |
| 26 | POLE2 | "DNA polymerase epsilon 2, accessory subunit [Source:HGNC Symbol;Acc:HGNC:9178]" | 4237 | 0.134 | 0.4068 | No |
| 27 | ERCC5 | "ERCC excision repair 5, endonuclease [Source:HGNC Symbol;Acc:HGNC:3437]" | 4380 | 0.122 | 0.4032 | No |
| 28 | MNAT1 | MNAT1 component of CDK activating kinase [Source:HGNC Symbol;Acc:HGNC:7181] | 4681 | 0.098 | 0.3857 | No |
| 29 | GTF2H4 | general transcription factor IIH subunit 4 [Source:HGNC Symbol;Acc:HGNC:4658] | 6295 | -0.027 | 0.2619 | No |
| 30 | POLD2 | "DNA polymerase delta 2, accessory subunit [Source:HGNC Symbol;Acc:HGNC:9176]" | 6311 | -0.028 | 0.2624 | No |
| 31 | CUL4A | cullin 4A [Source:HGNC Symbol;Acc:HGNC:2554] | 6701 | -0.055 | 0.2355 | No |
| 32 | LIG1 | DNA ligase 1 [Source:HGNC Symbol;Acc:HGNC:6598] | 6944 | -0.075 | 0.2212 | No |
| 33 | POLD3 | "DNA polymerase delta 3, accessory subunit [Source:HGNC Symbol;Acc:HGNC:20932]" | 7568 | -0.125 | 0.1802 | No |
| 34 | ERCC6 | "ERCC excision repair 6, chromatin remodeling factor [Source:HGNC Symbol;Acc:HGNC:3438]" | 7939 | -0.150 | 0.1605 | No |
| 35 | ERCC3 | "ERCC excision repair 3, TFIIH core complex helicase subunit [Source:HGNC Symbol;Acc:HGNC:3435]" | 8669 | -0.206 | 0.1162 | No |
| 36 | RAD23B | "RAD23 homolog B, nucleotide excision repair protein [Source:HGNC Symbol;Acc:HGNC:9813]" | 8777 | -0.215 | 0.1207 | No |
| 37 | RAD23A | "RAD23 homolog A, nucleotide excision repair protein [Source:HGNC Symbol;Acc:HGNC:9812]" | 9028 | -0.234 | 0.1153 | No |
| 38 | GTF2H1 | general transcription factor IIH subunit 1 [Source:HGNC Symbol;Acc:HGNC:4655] | 9455 | -0.269 | 0.0984 | No |
| 39 | RFC5 | replication factor C subunit 5 [Source:HGNC Symbol;Acc:HGNC:9973] | 9499 | -0.274 | 0.1115 | No |
| 40 | POLE | "DNA polymerase epsilon, catalytic subunit [Source:HGNC Symbol;Acc:HGNC:9177]" | 11026 | -0.433 | 0.0189 | No |
| 41 | GTF2H3 | general transcription factor IIH subunit 3 [Source:HGNC Symbol;Acc:HGNC:4657] | 12001 | -0.593 | -0.0213 | No |
| 42 | RPA4 | replication protein A4 [Source:HGNC Symbol;Acc:HGNC:30305] | 12406 | -0.709 | -0.0101 | No |
| 43 | GTF2H2 | general transcription factor IIH subunit 2 [Source:HGNC Symbol;Acc:HGNC:4656] | 12612 | -0.806 | 0.0224 | No |
Table: GSEA details [plain text format]

  

Fig 2: KEGG\_NUCLEOTIDE\_EXCISION\_REPAIR      
 Blue-Pink O' Gram in the Space of the Analyzed GeneSet

  

Fig 3: KEGG\_NUCLEOTIDE\_EXCISION\_REPAIR: Random ES distribution      
 Gene set null distribution of ES for **KEGG\_NUCLEOTIDE\_EXCISION\_REPAIR**
